# Supplementary material for: Characterization of Schistosome Sox Genes and Identification of a Flatworm Class of Sox Regulators
Source: Pathogens. 2023 May 9;12(5):690. doi: 10.3390/pathogens12050690 (PMC10222431; doi:10.3390/pathogens12050690)
Supplement: Supplementary file 1 [file pathogens-12-00690-s001.zip › pathogens-2389168-supplementary.pdf]

```
CLUSTAL multiple sequence alignment by MUSCLE (3.8)

MmSOX2_NP_035573                -MYNMMETELKPPGPQQASGGGGGGGNATAAATGGNQKNSPDRVKRPMNAFMVWSRGQRR
HsSOX-2_NP_003097.1             -MYNMMETELKPPGPQQTS--GGGGGNSTAAAAGGNQKNSPDRVKRPMNAFMVWSRGQRR
SmSoxS1_Smp_301790.3            MQSNLANNHL----------------------TVCNNNMNSERIKRPMNAFMVWSRLQRR
                                   *: :. *                      :  *:: ..:*:************ ***

MmSOX2_NP_035573                KMAQENPKMHNSEISKRLGAEWKLLSETEKRPFIDEAKRLRALHMKEHPDYKYRPRRKTK
HsSOX-2_NP_003097.1             KMAQENPKMHNSEISKRLGAEWKLLSETEKRPFIDEAKRLRALHMKEHPDYKYRPRRKTK
SmSoxS1_Smp_301790.3            KMAEENPKLHNSEISKQLGNLWKSLTNTDKIPFIEEANRLRDCHMRRYPNYKYCPRRKRK
                                ***:****:*******.**  ** *::*:* ***:**:***  **. :*:*** **** *

MmSOX2_NP_035573                TLMK--------KDKYTLPGGLLAPGGNSMASGVGVGAGLGAGVNQRMDS------YAHM
HsSOX-2_NP_003097.1             TLMK--------KDKYTLPGGLLAPGGNSMASGVGVGAGLGAGVNQRMDS------YAHM
SmSoxS1_Smp_301790.3            QPTNKVVLQHKVKTTSELPLELVLCPTRSNCFQASRLTRVSKVVTQPFNKPRIPIIAPTM
                                   :        * .  **  *:    .* .  ..  : :.  *.* ::.       . *

MmSOX2_NP_035573                NGWS-NGSYSMMQEQLGYPQHPGLNAHGAAQMQPMHRYDVSALQYNSMTSSQTYMNGSPT
HsSOX-2_NP_003097.1             NGWS-NGSYSMMQDQLGYPQHPGLNAHGAAQMQPMHRYDVSALQYNSMTSSQTYMNGSPT
SmSoxS1_Smp_301790.3            KSYEITQNRNMFVNEMNHKPEP-FIITSQMNMMPMS----NKLMPNICTSVQNLMADNNS
                                :.:. . .*: :::.:   * :   .  :* **     . *  *  ** *. * .. :

MmSOX2_NP_035573                YSMSYSQQGTPGMALGSMGSVVKSEASSSPPVVTSSSHSRAPCQAGDLRDMISMYLPGAE
HsSOX-2_NP_003097.1             YSMSYSQQGTPGMALGSMGSVVKSEASSSPPVVTSSSHSRAPCQAGDLRDMISMYLPGAE
SmSoxS1_Smp_301790.3            AYLQSTD---------TIYDPYKTQLSST--FLHNVNNQHNITNYPDQKEFYTDF----N
                                  :. ::         :: .  *:: **:  .: . .:..   :  * .:: : :    :

MmSOX2_NP_035573                VPEPAAPSRLHMAQH----YQSGPVPG--TAINGTLPLSHM-
HsSOX-2_NP_003097.1             VPEPAAPSRLHMSQH----YQSGPVPG--TAINGTLPLSHM-
SmSoxS1_Smp_301790.3            VCQPTTSNDVNNCNHELCPYDITHSPEIYSSFLDSIDLQTFF
                                * :*::.. :: .:*    *:    *   ::: .:: *. :
```

Figure S1. MUSCLE alignment of mouse and human Sox2 (MmSOX2 and HsSOX-2) and schistosome SoxS1. The HMG domain is located in the red box.

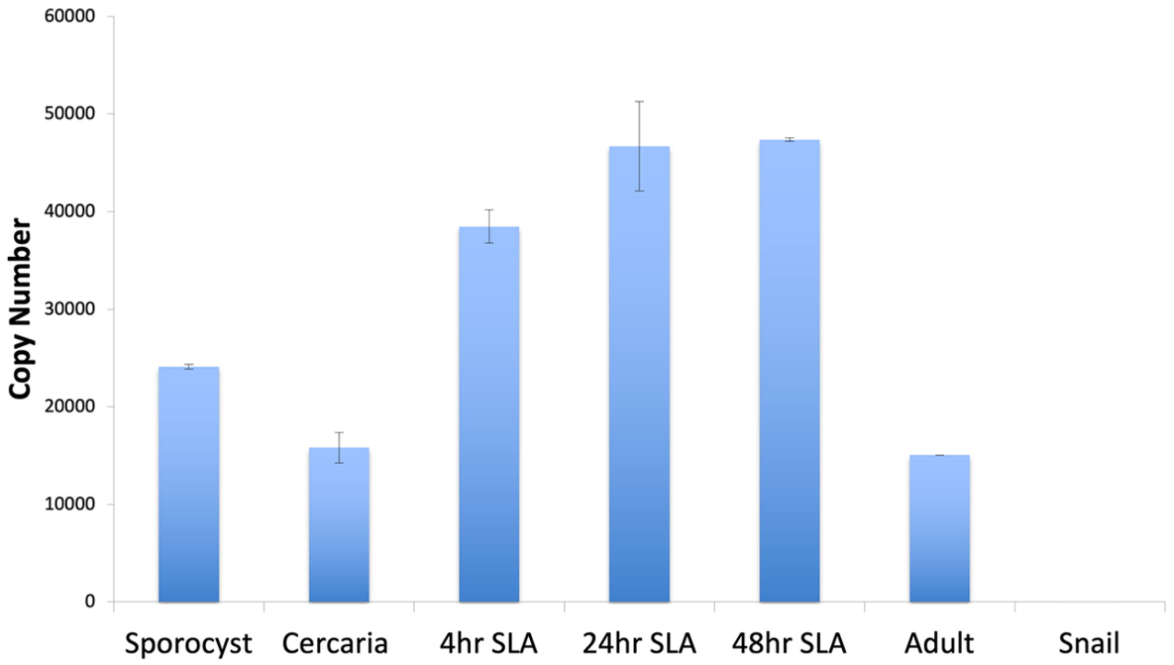

Figure S2. Absolute quantitative PCR for the schistosome cyclophilin transcript during schistosome development.
